# Iron: an underrated factor in aging

**DOI:** 10.18632/aging.203612

**Published:** 2021-10-06

**Authors:** Dennis Mangan

**Affiliations:** 1 MTOR LLC, Bakersfield, CA 93311, USA

**Keywords:** iron, aging, oxidative stress, calorie restriction, plasma dilution

## Abstract

Iron is an essential element for virtually all living organisms, but its reactivity also makes it potentially harmful. Iron accumulates with aging, and is associated with many age-related diseases; it also shortens the lifespans of several model organisms. Blocking iron absorption through drugs or natural products extends lifespan. Many life-extending interventions, such as rapamycin, calorie restriction, and old plasma dilution can be explained by the effects they have on iron absorption, excretion, and metabolism. Control of body iron stores so that they remain in a low normal range may be an important, lifespan- and healthspan-extending intervention.

All life forms require the element iron as a constituent of their biochemical systems, iron being used in producing ATP in mitochondria, in cytochromes and hemoglobin, and in many other uses. Iron is essential for organismal growth and maintenance, so all life, from bacteria and algae to mammals, have developed the means to collect and store iron from their environments; this centrality of iron for all life suggests that iron may be involved in aging. Most organisms, including humans, have no systematic means of ridding themselves of excess iron. Whether this lack of ways to dispose of excess iron came about due to a relative scarcity of iron, or because the detrimental results from excess iron were relatively rare in an environment in which few organisms died from natural aging, is a question that remains to be answered. Whatever the answer to that may be, most organisms accumulate iron as they age [1–3].

A problem that organisms face in the use of iron in biological systems is protecting cells from iron damage. The very property of iron that makes it useful, its ability to accept or donate electrons, also gives it the ability to damage molecules and organelles via the Fenton reaction, in which iron reacts with hydrogen peroxide, leading to the formation of the highly reactive and toxic free radical, hydroxyl.

Most iron in cells is bound to proteins and other molecules that safely store it and prevent it from interacting with other macromolecules. In mammals, ferritin and transferrin are such proteins; hemoglobin is, however, the quantitatively most important iron depot in mammals. In theory, these storage proteins should be enough to protect organelles and macromolecules from iron’s reactivity, but in practice another process becomes perhaps more important, and that is iron dysregulation. Storage proteins such as ferritin can themselves be damaged, leading to “leakage” of free iron, which can then react with and damage cellular structures, which in turn can lead to organ damage and the deterioration associated with aging [4]. Damage to ferritin can be caused by glycation due to hyperglycemia, a phenomenon seen more widely with the development of advanced glycation end products (AGEs), and with the glycation of hemoglobin (HbA1c), elevated in diabetics. The superoxide anion can also damage ferritin, leading to a vicious cycle in which leakage of free iron leads to oxidative stress, in turn leading to more iron leakage [5].

Whether this damage associated with aging is in fact a cause or consequence of aging of course remains to be determined, but as we shall see, there are several other reasons to think that iron is a driver of aging.

## Activation of mTOR

The mechanistic (or mammalian) target of rapamycin (mTOR), a molecular sensor that integrates nutritional and stress signals with growth and energy needs of the cell, plays a crucial role in aging; inhibition of mTOR with the drug rapamycin extends lifespan and healthspan in laboratory animals [6]. Nutritional and other factors that promote mTOR activation promote accelerated aging, and their absence may retard aging. For example, calorie restriction, the most robust life extension paradigm, also inhibits mTOR activation, while in humans, obesity resulting from overnutrition is a well-known cause of early morbidity and mortality [7]. In general, growth factors, whether amino acids, glucose, or fatty acids, or hormonal signals that they engender, such as insulin, promote mTOR activation, while their absence, or the presence of stress from exercise or other sources that activate the cellular energy sensor AMPK, inactivate mTOR.

Iron is one such growth factor. Iron is required for the growth of the organism, and iron activates mTOR; iron chelators, chemicals that bind free iron, inhibit mTOR activity [8]. Iron deficiency downregulates mTOR activity [9]. These data fit the paradigm of increased mTOR activity and aging, which may be promoted by excess iron.

In turn, mTOR also exerts control over iron metabolism, and the inhibition of mTOR activity by rapamycin leads to inhibition of iron accumulation via the iron-regulating hormone hepcidin [10]. Transplant patients taking sirolimus (rapamycin) often develop a microcytic anemia, which has been linked to sirolimus-induced iron deficiency [11].

Excessive activation of mTOR is seen in type 2 diabetes, and this activation is associated with insulin resistance [12]. mTOR activation in diabetes may be responsible for the accumulation of excess iron seen in this illness; alternatively, accumulation of iron might activate mTOR, leading to diabetes. Increased iron stores predict the development of type 2 diabetes, while iron depletion can protect against it [13]. Insulin resistance is associated with inadequate levels of hepcidin, an iron regulatory hormone, which could be expected to increase body iron stores [14]. So there’s evidence that iron increases insulin resistance, and that in turn can lead to higher body iron in a vicious cycle. Since type 2 diabetes is an age-related disease, it can be seen how excess iron promotes aging.

However, even when ferritin is in the normal range, depletion of iron improves glucose tolerance, insulin resistance, and markers of cardiovascular disease [5]. Iron appears to have a dose-response effect starting from near iron deficiency up to iron overloading, making it a candidate driver of aging.

## Blocking iron extends lifespan

In experimental organisms and animals, blocking iron extends lifespan.

In *Saccharomyces cerevisiae*, limitation of iron increases chronological lifespan via inducing autophagy [15]. Autophagy is essential for lifespan extension, so this may be the ultimate means by which iron restriction or depletion extends lifespan, and iron excess promotes aging [16].

Dietary tea extracts increase the lifespan of *Drosophila* by over 20% by blocking the absorption of iron [17].

A number of geroprotectors increase lifespan in model organisms, and many of these either block dietary iron absorption or chelate iron and remove it.

Curcumin and its metabolite tetrahydrocurcumin increase average lifespan in at least three model organisms: *C. elegans, Drosophila*, and mice [18]. Curcumin is a strong iron chelator; animals fed curcumin had a decline in liver ferritin [19]. Mice fed 0.2% curcumin in the diet become iron deficient; levels of zinc and copper were not affected [20].

Epigallocatechin gallate (EGCG), a compound found in green tea, extends lifespan of both *C. elegans* and *Drosophila* [21, 22].

EGCG extends the lifespan and healthspan of mice, attenuating markers of DNA damage and senescence-associated secretory profile, and increasing activation of autophagy [23]. EGCG also extends the lifespan of rats by reducing liver and kidney damage and inhibiting inflammation and oxidative stress [24].

EGCG is a strong chelator of iron [25]. EGCG protects against alcoholic liver disease in mice through decreasing the level of liver iron [26].

Aspirin extends the lifespan of *C. elegans* [27] and that of mice [28]. In humans, aspirin reduces the risk of cancer [29].

Aspirin use is associated with lower body iron stores, perhaps through an increase in gastrointestinal blood loss; observational studies have shown that regular aspirin users have lower serum ferritin; as cancer cells are notoriously iron-hungry, this might partially explain the reduced cancer risk with aspirin [30]. Aspirin also recapitulates several features of calorie restriction, which could be expected to result in lower levels of body iron [31]. Salicylate, the main metabolite of aspirin, forms a complex with iron, and this process can be used in the quantitative detection of salicylate [32]. Bacteria elaborate siderophores in order to capture iron from their environment, and one such siderophore seen in several species of *Pseudomonas* is salicylate [33]. Aspirin increases the synthesis of ferritin in endothelial cells, which would result in lower levels of free iron, providing an antioxidant function; aspirin failed to promote ferritin synthesis in the presence of the iron chelator deferoxamine, indicating an interaction of aspirin and iron [34]. All of these data fit well with the idea that aspirin extends lifespan and inhibits cancer through decreasing body iron as at least one mechanism.

A screening of drugs for protection against neuronal glucose toxicity found six of them that reduce mortality rate in *C. elegans*: caffeine, tannic acid, ciclopirox, acetaminophen, bacitracin, and baicalein [35]. Of these, with the possible exception of caffeine, all chelate iron. Caffeine has weak iron-binding ability [36]. Tannic acid, ciclopirox, bacitracin, and baicalein are strong iron chelators [37–40]. Acetaminophen protects against iron-induced cardiac damage in gerbils [41]. Thus there is evidence that a primary mechanism of these life-extending compounds is the binding of free iron and protection against oxidant-induced damage.

Clofibrate increases lifespan in *C. elegans* [42]. When fed to Wistar rats, clofibrate led to a 50% decrease in serum iron and a reduction in transferrin mRNA [43].

Therefore, this is yet another example of a life-extending drug, the mechanism of which may at least partially involve decreased iron stores.

Berberine extends lifespan in mice [44]. Berberine also suppresses gero-conversion [45]. Berberine also has “a marked capacity” for iron-binding, and effectively chelates iron [46].

Acarbose extends lifespan in mice [47]. Acarbose increases fecal excretion of iron and has been known to be a cause of iron-deficiency anemia in humans [48, 49].

Doxycycline extends lifespan in *C. elegans* [50]. Doxycycline has a “strong iron-chelating activity” [51].

Enalapril is an angiotensin converting enzyme inhibitor that increases lifespan in rats [52]. One of the adverse effects of ACE inhibitors in humans is a dry cough, which is relieved by iron administration, indicating that one effect of these drugs concerns iron metabolism [53].

Ibuprofen extends lifespan in at least three organisms: *Saccharomyces cerevisiae, C. elegans,* and *Drosophila* [54]. Ibuprofen chelates iron and protects against oxidant lung injury by this means [55].

Metformin increases lifespan and healthspan in mice [56]. Many mechanisms have been proposed for the effects of metformin. One such mechanism is that, at concentrations seen in clinical use, metformin suppresses heme production in human erythrocytes, and prevents heme oxidation, thus having a role in regulating the redox status of iron [57]. In yeast, a global genetic screen showed that metformin induces a state similar to iron deficiency [58].

Quercetin, a polyphenol found in food, extends lifespan in *C. elegans*, and it appears to do so by increasing resistance to oxidative stress [59]. Quercetin is “a powerful chelating agent that can sequester iron(II) in such a way to prevent its involvement in the Fenton reaction [60].”

Thus, we can see that a large number of life-extending compounds also interact with iron, either by chelation, inhibition of absorption, or increased iron loss.

## Calorie restriction

Calorie restriction (CR) is the most robust life-extending intervention known. Many mechanisms have been proposed to explain lifespan extension by CR, such as its effects on insulin and IGF-1 signaling, mTOR, sirtuins, AMPK, adiposity, and resistance to oxidative stress [61]. CR also affects iron metabolism.

In yeast grown on a low-glucose medium, which is a model of CR, oxidative damage in the form of protein carbonylation is largely prevented. Intracellular iron concentrations changed little, whereas in yeast cells grown on non-restrictive media, iron concentrations increased up to 5-fold. The pro-oxidant effects of these increasing iron concentrations might explain the molecular damage seen in unrestricted cells, and the lower iron seen in CR yeast might explain the lower levels of damage. Thus, lower levels of iron in CR yeast can be posited as an important mechanism of increased longevity in CR [62].

CR could also be expected to result in lower ingestion of iron. When iron is the only limiting nutrient, yeast chronological lifespan is extended through induction of autophagy, which is essential for increased longevity [15].

CR was found to substantially decrease the increase in liver, kidney, and brain iron seen in rats fed ad libitum. Lipid peroxidation was also markedly suppressed in CR animals. Thus, CR has an antioxidant effect which may be largely due to decreased levels of body iron [63].

CR downregulates expression of the iron-regulatory hormone hepcidin in the brain, and this leads to less accumulation of brain iron in aging, which is a key component of neurodegenerative diseases [64]. CR leads to less brain iron deposition in old rhesus monkeys, along with preserved motor performance [65].

Attenuation of increasing iron in liver, kidney, brain, and other tissues may be an important mechanism of the longevity-promoting effects of CR.

Increased dietary iron promotes protein insolubility and aging in *C. elegans*, while pharmacological intervention to block uptake of iron mitigated much damage and extended normal lifespan [66].

## Heterochronic blood exchange, plasma dilution, and blood donation

Heterochronic blood exchange between young and old mice results in “rapid inhibition of multiple tissues by old blood”, for reasons that are not clear [67]. Since old animals accumulate iron, and since they exhibit more iron dysregulation resulting in higher levels of free iron, iron may be suspected as the mechanism behind this inhibition of younger tissues by old blood.

In old animals, mere plasma dilution by exchanging it with saline and 5% albumin (an age-neutral blood exchange) leads to rejuvenation of muscle, liver, and brain in old mice [68]. Since there is no young blood involved in this exchange, this makes it doubtful that factors in young blood play an important (if any) role in rejuvenation seen in heterochronic blood exchange.

Human serum from patients who undergo therapeutic plasma exchange (TPE) was also tested for its ability to rejuvenate cells. Old human serum strongly reduced proliferation of mouse myogenic cells, while a single TPE from the same patients reversed this inhibition. Accumulation of iron delays muscle regeneration and suppresses the differentiation of myoblast cells, and this suppression can be reversed with superoxide scavenging [69].

If TPE can lead to serum becoming (or reverting to) a rejuvenating intervention, the question is, what was removed from the serum that allowed for this effect?

Iron makes a good candidate. In TPE, citrate may be used as an anticoagulant; citrate complexes with free iron, and the citrate-iron complex is the major species of iron found in iron overloaded patients [70]. Patients undergoing TPE have a high rate of iron deficiency anemia; one study found that 60% of patients developed iron deficiency anemia, and 100% of patients had decreased serum iron [71]. This may be due to use of citrate as an anticoagulant, or due to simple removal of plasma, which contains transferrin, and replacement with an albumin solution [72]. Other components of plasma may be removed or diluted as well, but iron may be the critical element here.

Blood donation leads to lower levels of body iron; hemoglobin is the main iron depot in the body, hence its replacement after donation requires the use of body iron stores and decreases them. Several studies have found lower mortality in blood donors, even after accounting for a healthy donor effect from donors being healthier than others even before donation. When only blood donors are studied as a single class, there is an inverse association between blood donation frequency and mortality, with each additional annual donation associated with an 7.5% reduced mortality rate [73]. Blood donation is associated with a marked decrease of body iron in adult men; ferritin values of <15 μg/L (depleted) are about 8 times more common in male donors than in non-donors, and iron deficiency anemia is up to 5 times more common in donors than non-donors [74].

## Conclusion: iron squares the circle of life extension

It can be seen from all of the above that iron is a common theme in many if not most life-extension interventions. This can help make sense of the seemingly disparate mechanisms of extending life by slowing aging.

As noted, autophagy is essential for lifespan extension, and autophagy activation declines with age. Ultimately, this can lead to “the garbage catastrophe of aging”, in which imperfect removal of damaged molecules leads to the accumulation of cellular “garbage”. Much of this decline in autophagy may be due to lipofuscin, a substance that is relatively difficult to remove and which “gums up” the machinery of autophagy. Importantly, iron plays a key role in the formation of lipofuscin; iron can react with polyunsaturated fatty acids and other molecules to form this material, and iron accelerates lipofuscin formation in cultured human glial cells and rat cardiomyocytes [75]. Lower levels of iron could be expected to slow the rate of lipofuscin formation.

Inhibiting the cellular integrator of nutrients and growth, mTOR, leads to longer lifespan in virtually all experimental animals tested so far. We have seen that mTOR in turn plays a crucial role in the level of body iron stores; mTOR activation increases body iron, and iron in turn activates mTOR. That mTOR inhibition increases lifespan illustrates the fundamental trade-off between growth and longevity, and iron is a growth factor [76].

Many drugs and natural products extend lifespan by seemingly disparate mechanisms, but inhibiting iron absorption, or chelating (binding and removing) iron is a characteristic of many if not most of these substances.

Reduced iron stores can explain how calorie restriction extends lifespan.

Finally, iron can explain the free radical theory of aging. Iron catalyzes the formation of the most damaging free radical, the hydroxyl radical.

In sum, iron satisfies many of the conditions we might look for in a universally pro-aging substance. It accumulates with age; it is associated with many age-related diseases such as cardiovascular disease, cancer, and Alzheimer’s disease; it catalyzes the formation of cellular junk molecules and helps to prevent their turnover; removal of iron from plasma may be rejuvenating; and people with lower levels of body iron – blood donors – have a lower mortality rate.

Iron is intimately associated with aging, and control of body iron stores may be an important way to extend human lifespan.
